# Sphingosine 1-phosphate receptor 2 (S1P_2_) attenuates reactive oxygen species formation and inhibits cell death: implications for otoprotective therapy

**DOI:** 10.1038/srep24541

**Published:** 2016-04-15

**Authors:** Deron R. Herr, Marie J. Y. Reolo, Yee Xin Peh, Wei Wang, Chang-Wook Lee, Rich Rivera, Ian C. Paterson, Jerold Chun

**Affiliations:** 1 Department of Pharmacology, Yong Loo Lin School of Medicine, National University of Singapore, Singapore 117597; 2 Department of Biology, San Diego State University, San Diego, CA, USA; 3 Department of Molecular and Cellular Neuroscience, Dorris Neuroscience Center, The Scripps Research Institute, La Jolla, CA, USA; 4 Department of Oral Biology and Biomedical Sciences and Oral Cancer Research & Coordinating Centre, Faculty of Dentistry, University of Malaya, Kuala Lumpur, Malaysia

## Abstract

Ototoxic drugs, such as platinum-based chemotherapeutics, often lead to permanent hearing loss through apoptosis of neuroepithelial hair cells and afferent neurons of the cochlea. There is no approved therapy for preventing or reversing this process. Our previous studies identified a G protein-coupled receptor (GPCR), S1P_2_, as a potential mediator of otoprotection. We therefore sought to identify a pharmacological approach to prevent cochlear degeneration via activation of S1P_2_. The cochleae of *S1pr2*^−/−^ knockout mice were evaluated for accumulation of reactive oxygen species (ROS) with a nitro blue tetrazolium (NBT) assay. This showed that loss of S1P_2_ results in accumulation of ROS that precedes progressive cochlear degeneration as previously reported. These findings were supported by *in vitro* cell-based assays to evaluate cell viability, induction of apoptosis, and accumulation of ROS following activation of S1P_2_ in the presence of cisplatin. We show for the first time, that activation of S1P_2_ with a selective receptor agonist increases cell viability and reduces cisplatin-mediated cell death by reducing ROS. Cumulatively, these results suggest that S1P_2_ may serve as a therapeutic target for attenuating cisplatin-mediated ototoxicity.

Hair cells of the cochlea are specialized neuroepithelial cells required for the transduction of vibrational force into the perception of hearing. In mammals, these post-mitotic, terminally differentiated cells are fully developed shortly after birth, thus they do not have the capacity to regenerate if they are lost. There are 3 major causes of acquired (non-hereditary) sensorineural hearing loss: noise exposure, ototoxicity, and age. These causes account for about half of the estimated 700 million cases of debilitating hearing loss worldwide^1^. Major classes of ototoxic drugs include chemotherapeutics (cisplatin), aminoglycoside antibiotics (kanamycin), and loop diuretics (furosemide). While the molecular events leading to ototoxicity are complex, there is evidence that they are, at least in part, mediated by toxic accumulation of reactive oxygen species (ROS). The NOX family of NADPH oxidases (NOX1-NOX5 and DUOX1–2) is a major source of endogenous ROS formation^2^. Its members are multi-subunit, membrane-associated enzymatic structures with complex regulatory machinery. One family member, NOX3, is highly and selectively expressed in the inner ear, with little detectable expression in other tissues^3^.

Despite information regarding the molecular events underlying cochlear degeneration, there are currently no therapies that can prevent or reverse this process. This is due in large part to the absence of pharmacologically tractable molecular targets that regulate cochlear viability. Previous work by our group and others provide evidence that S1P_2_, a G protein-coupled receptor (GPCR) that mediates the effects of sphingosine 1-phosphate (S1P), may represent such a target.

S1P is a bioactive lipid signalling molecule that is known to act as a potent extracellular ligand for a family of five cognate GPCRs, S1P_1_–S1P_5_^4^. These receptors have distinct but overlapping patterns of expression, and are known to be important activators of many cellular processes, such as cell proliferation, cell death, cytoskeletal rearrangement, migration/motility, and differentiation^5^,^6^. Notably, receptor-mediated S1P signalling has been shown to affect the production of ROS in the heart^7^, blood vessels^8^, fibroblasts^9^, and hematopoietic progenitor cells^10^.

Many of the biomedically relevant roles of S1P receptors have been elucidated with the study of genetically engineered knockout mice. These studies have shown that S1P signalling is essential for a number of processes including vascular maturation^11^, lymphocyte trafficking^12^, epithelial sheet migration^13^, B cell regulation^14^, egress of natural killer cells^15^, and mechanisms underlying the multiple sclerosis drug known as fingolimod (Gilenya)^16^,^17^,^18^. Recently, it was shown that S1P_2_ knockout mice uniformly exhibit a progressive loss of inner ear function, resulting in profound deafness and vestibular dysfunction, demonstrating that S1P_2_ activity is necessary for cochlear viability^19^,^20^,^21^ that was a ligand-dependent process since loss of S1P transporter gene *Spns2* phenocopies S1P_2_ loss^22^. Cochlear degeneration was associated with early vascular defects that likely alter cochlear perfusion pressure and disrupt electrochemical gradients required for hair cell function^20^. While there is strong evidence that this mechanism contributes to loss of cochlear integrity, we note that S1P_2_ is expressed in the hair cells and supporting cells of the cochlea, with expression increasing over time, coincident with the progression of the cochlear degeneration^19^. Therefore, we examined the possibility that additional, cell-intrinsic functions of S1P_2_ promote viability of cochlear structures.

The recent approval of fingolimod, a non-selective functional antagonist of four S1P receptors, for the treatment of multiple sclerosis^23^ demonstrates the feasibility of developing small molecule drugs that target S1P receptor signalling. Considering the pleotropic functions of the S1P receptor family, it would be a significant advantage to develop subtype-selective ligands as drug candidates. In this study, we demonstrate that activation of S1P_2_ is associated with reduction of ROS accumulation by a specific S1P_2_ agonist and provide proof-of-concept for its use as an otoprotective agent.

## Results

### Loss of S1P_2_ results in ROS generation and cochlear degeneration

**Figure 1:**
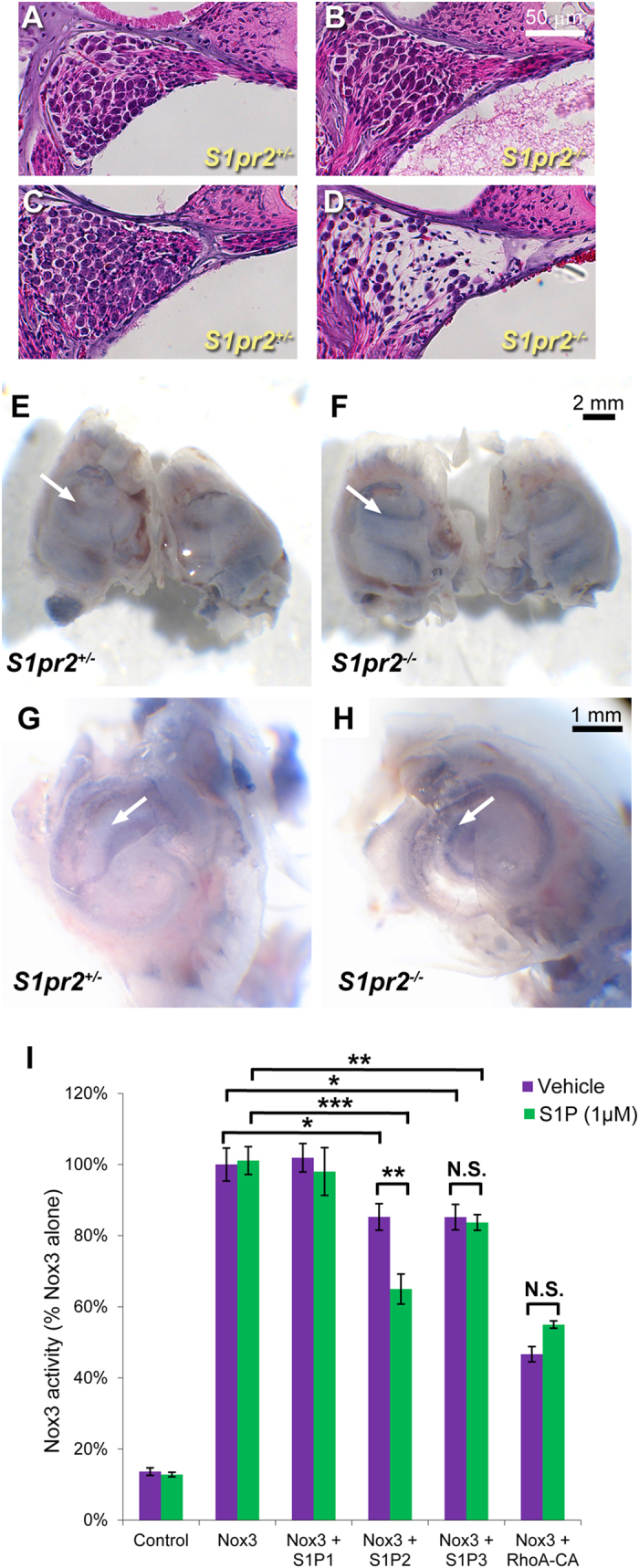
S1P_2_ attenuates ROS accumulation in the cochlea. Hematoxylin and eosin staining of cochlear tissue sections demonstrates structurally intact neurons in the spiral ganglia of *S1pr2*^+/−^ (**A**) and *S1pr2*^−/−^ (**B**) mice at 2 weeks of age. By 8 weeks of age, *S1pr2*^+/−^ cochlea remain intact (**C**), but spiral ganglia of *S1pr2*^−/−^ mice (**D**) demonstrate marked, progressive degeneration. (Results previously described^19^). The NBT assay demonstrates little staining in *S1pr2*^+/−^ cochlea (**E**), but a consistent banding pattern in *S1pr2*^−/−^ littermate cochlea (**F**), indicative of ROS accumulation. (**G,H**) Higher magnification reveals that the most intense staining is localized to the spiral ganglia. Images are representative of 7–9 mice per genotype. (**I**) *In vitro* assay for recombinant NOX3 activity reveals that S1P_2_, but not S1P_1_ or S1P_3_, can inhibit NOX3 activity in a ligand dependent manner. Co-transfection with constitutively active Rho, a known downstream mediator of S1P_2_ signalling, demonstrates a similar, but ligand-independent, inhibitory effect on NOX3. Scale bars represent 50 μm (**A–D**), 2 mm (**E,F**), and 1 mm (**G,H**), respectively. (*p < 0.05, **p < 0.01, ***p < 0.001).

We previously reported that *S1pr2*^−/−^ knockout mice exhibit progressive degeneration of the sensory structures of the cochlea and vestibular end organs^19^. This is particularly evident in the spiral ganglia that innervate the organ of Corti. At 2 weeks of age, the neurons of the spiral ganglia are intact and indistinguishable from those of wild-type mice (Fig. 1A,B), but as previously described^19^, by 8 weeks of age there is marked degeneration of the ganglia at the basal turn of the cochlea, characterized by pronounced neuronal loss (Fig. 1C,D). To determine whether accumulation of ROS may contribute to this process, we evaluated wild-type and knockout cochlea for ROS content at 3 weeks of age prior to the onset of frank degeneration. Cochlea derived from *S1pr2*^−/−^ mice were characterized by the formation of dense blue labeling in the area of the spiral ganglia following incubation with NBT, indicating accumulation of ROS in the afferent nerve fibers. By contrast, specific labeling of the spiral ganglia was absent in *S1pr2*^+/−^ littermates (Fig. 1E–H).

To explain how loss of *S1pr2* may lead to ROS accumulation, we sought to determine whether S1P_2_ could regulate the activity of NOX3^24^,^25^. Transfection of HEK293 cells with the NOX3 complex resulted in a marked increase in ROS generation (Fig. 1I). This could be significantly attenuated by co-transfection with S1P_2_, and further attenuated by activation of S1P_2_ with S1P (1 μM). S1P-dependent reduction of ROS was not observed upon co-transfection with either of two other S1P receptor subtypes, S1P_1_ and S1P_3_. NOX3 activity was also similarly inhibited by co-transfection with constitutively-active RhoA (RhoA-CA), which is known to act downstream of S1P_2_^26^. The use of RhoA-CA provides a useful control condition in that it 1) confirms that S1P_2_-regulated pathways inhibit NOX3, and 2) demonstrates the maximum inhibitory response that would be expected from activation of the RhoA pathway.

### CYM-5478 is a potent, selective agonist for S1P_2_

**Figure 2:**
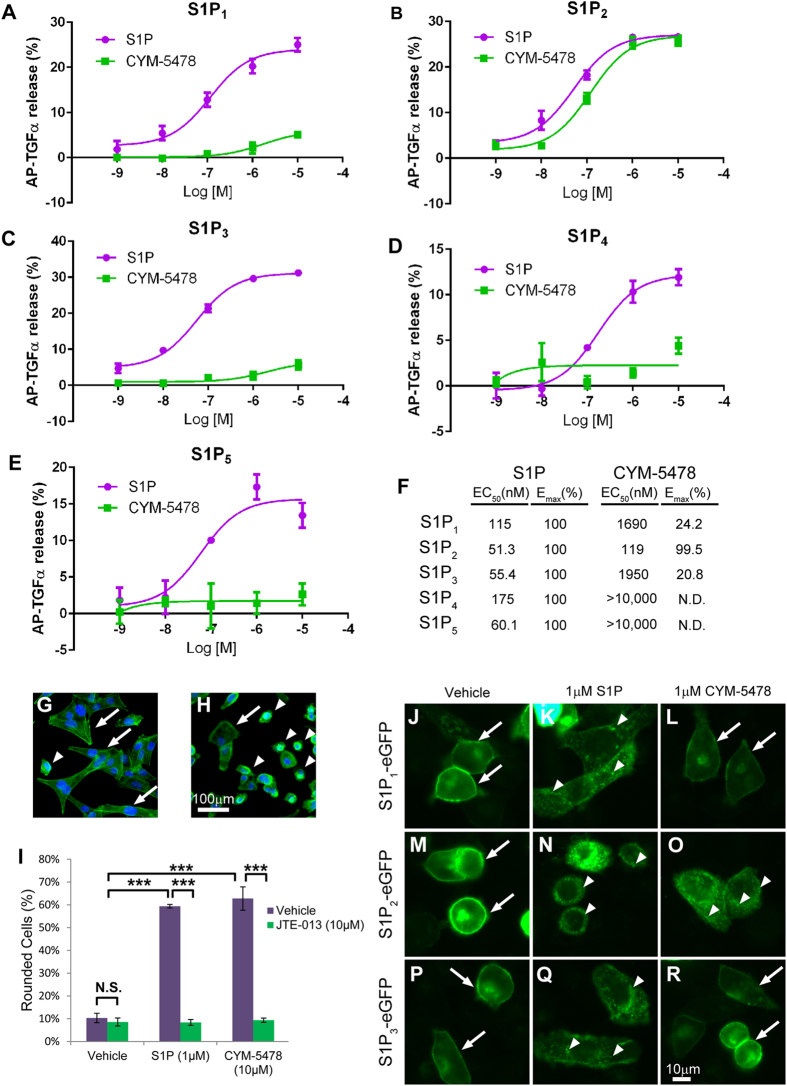
CYM-5478 is an S1P_2_-selective agonist. The TGFα-shedding assay was performed to evaluate the activation of S1P_1_ (**A**), S1P_2_ (**B**), S1P_3_ (**C**), S1P_4_ (**D**), and S1P_5_ (**E**) by CYM-5478 relative to that of endogenous ligand, S1P. (**F**) A summary of the TGFα-shedding results demonstrates that only S1P_2_ is potently and effectively activated by CYM-5478. (**G**) MDA-MB-231 breast cancer cells typically display an elongated, fibroblast-like morphology (arrows), with few rounded cells (arrowheads), as visualized by labelling with fluorescein-phalloidin. (**H**) Upon stimulation with 1 μM S1P MDA-MB-231 cells become rounded. (**I**) Quantitation of cell rounding demonstrates that the response to CYM-5478 is equivalent to that of S1P, and that the activity of both ligands can be inhibited by pre-treatment with S1P_2_ antagonist, JTE-013. (N = 3). S1P and CYM-5478 were evaluated for their ability to induce the internalization of S1P_1_-EGFP (**J**–**L**), S1P_2_-EGFP (**M**–**O**), and S1P_3_-EGFP (**P**–**R**) fusion proteins. (**J**,**M**,**P**) All receptors were predominantly localized to the plasma membrane (arrows) when treated with vehicle alone. (**K**,**N**,**Q**) All receptors were largely internalized to cytoplasmic vesicles (arrowheads) when stimulated with 1 μM S1P. (**L**,**O**,**R**) CYM-5478-treatment induced the internalization of S1P_2_-EGFP, but not S1P_1_-EGFP or S1P_3_-EGFP. Scale bars represent 100 μm (**G**,**H**) and 100 μm (**J**–**R**), respectively. (Images are representative of 3 independent experiments). (***p < 0.001).

To determine whether activation of S1P_2_ may be cytoprotective, we sought an S1P_2_-selective agonist. CYM-5478 was identified as a candidate S1P_2_ agonist in a high throughput screen by The Scripps Research Institute Molecular Screening Center (http://pubchem.ncbi.nlm.nih.gov/assay/assay.cgi?aid=872). This compound (PubChem CID: 7802604) had a reported EC_50_ of 723 nM in the original screen, and an EC_50_ of 780 nM in subsequent validation studies^27^. We performed several cell-based assays to confirm this result, and to evaluate the selectivity of CYM-5478. Use of a TGFα-shedding assay^28^ demonstrated that CYM-5478 activates S1P_2_ with an EC_50_ of 119 nM, but had less than 25% efficacy and showed 10-fold lower potency against the other S1P receptor subtypes (Fig. 2A–F). Control cells transfected with empty vector did not exhibit a measurable response when stimulated with either S1P or CYM-5478 (data not shown).

To confirm that the activation of S1P_2_ was not an artefact of receptor overexpression, we evaluated the ability of CYM-5478 to activate endogenously expressed S1P_2_ receptors in MDA-MB-231 breast cancer cells. Upon stimulation with S1P (1 μM), serum-starved MDA-MB-231 cells displayed pronounced cytoskeletal rearrangement characterized by process retraction and cell rounding (Fig. 2G,H). This response was completely abrogated by pre-treatment with S1P_2_-selective antagonist JTE-013^29^. CYM-5478 elicited an identical rounding response in MDA-MB-231 cells, which was similarly abrogated by JTE-013 (Fig. 2I).

Activation of S1P receptor-EGFP constructs results in their internalization into cytoplasmic vesicles that can be visualized by fluorescence microscopy^18^. We exploited this effect to further validate the use of CYM-5478 as an S1P_2_-specific agonist. Both S1P (1 μM) and CYM-5478 (1 μM) were able to induce the translocation of S1P_2_ from the plasma membrane to cytoplasmic vesicles, but only S1P was effective against S1P_1_ and S1P_3_ (Fig. 2J–R).

### CYM-5478 promotes viability and inhibits cell death in neural cells *in vitro*

**Figure 3:**
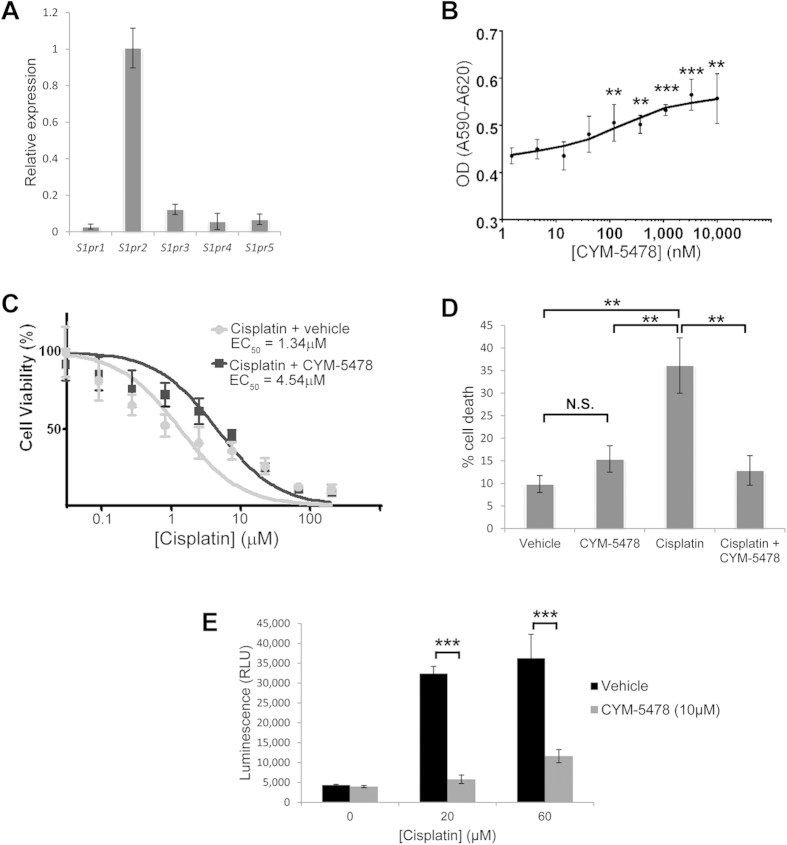
CYM-5478 promotes cell viability. (**A**) qRT-PCR demonstrates that S1P_2_ is the predominant S1P receptor expressed in C6 cells. (**B**) When treated with CYM-5478, C6 cells demonstrated a dose-dependent increase in viability, as evaluated by MTT assay. (N = 6. Asterisks indicate significant differences compared to 0 μM CYM-5478). (**C**) C6 cells exhibit a dose-dependent decrease in viability when treated with cisplatin. Co-administration of 10μM CYM-5478 reduces sensitivity to cisplatin and confers a 3-fold increase in EC_50_. (**D**) C6 cells exhibit a >3-fold increase in propidium iodide-positive cells when treated with cisplatin. This effect is attenuated by co-administration of CYM-5478. There is no significant change in propidium iodide-positive cells due to CYM-5478-treatment alone. (N = 8). (**E**) Cisplatin induces a significant increase in caspase 3/7 activity in C6 cells. This effect is markedly attenuated by co-administration of CYM-5478. (N = 3). (**p < 0.01, ***p < 0.001)).

Since *loss* of S1P_2_ results in progressive degeneration of sensory epithelial hair cells, supporting cells, and afferent neurons of the cochlea^19^, we sought to determine whether *activation* of S1P_2_ could promote viability of a neural-derived cell line. C6 cells rat glioma cells^30^ were evaluated by RT-PCR and shown to express S1P_2_ as its predominant S1P receptor subtype (Fig. 3A). Under nutrient-deprivation stress produced by serum-starvation, CYM-5478 induced a statistically significant increase in the viability of C6 cells in a dose dependent manner at concentrations above 100 nM (Fig. 3B). This effect was absent in the presence of 10% fetal bovine serum (data not shown) suggesting that the increase in viability was a result of decreased starvation-induced cell death, rather than an increase in proliferation.

Since cisplatin is a known ototoxic compound that exerts its action, at least in part, by increased NOX3 activity^3^, we sought to determine whether activation of S1P_2_ could protect cells from cisplatin-mediated death. In the presence of CYM-5478 (10 μM) there was a statistically significant, 3-fold increase in the EC_50_ of cisplatin-mediated reduction in the viability of C6 glioma cells (Fig. 3C), consistent with pronounced cytoprotection produced by S1P_2_ activation. Similarly, CYM-5478 treatment was able to abrogate cisplatin-induced cell death, as evaluated by propidium iodide dye exclusion assay (Fig. 3D). We further found that this effect was coincident with the reduction of apoptosis. Treatment with CYM-5478 also attenuated cisplatin-induced caspase 3/7 activity in C6 cells (Fig. 3E). Taken together, these data demonstrate a significant cytoprotective effect through the activation of S1P_2_ by CYM-5478.

### S1P_2_ activation inhibits the generation of ROS

**Figure 4:**
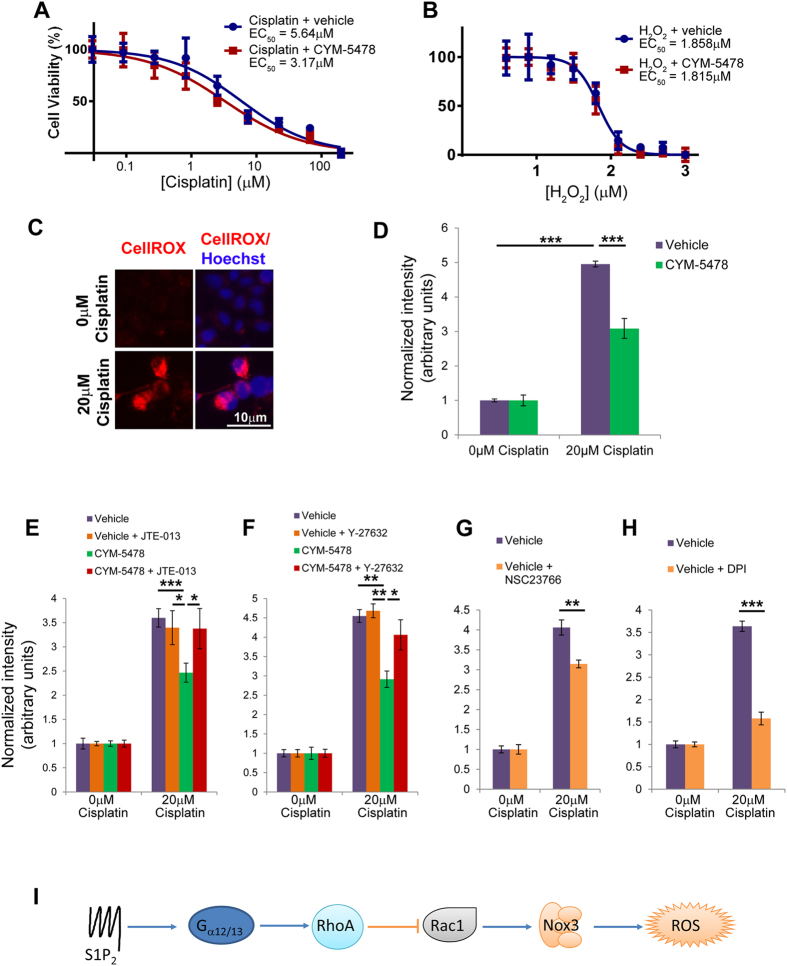
CYM-5478 acts via reduction of endogenous ROS. (**A**) In the presence of 1 mM N-acetylcysteine, there is no significant difference in the sensitivity of CYM-5478-treated C6 cells compared to vehicle-treatment alone. (N = 6). (**B**) There is no significant difference in the sensitivity of C6 cells to hydrogen peroxide in the presence of CYM-5478 compared to vehicle-treatment alone. (N = 6). (**C**) Cisplatin-treatment causes increased fluorescence intensity of C6 cells treated with CellROX® reagent, indicative of ROS accumulation. (**D**) Quantification of CellROX® fluorescence demonstrates that co-administration of CYM-5478 (10 μM) attenuates the cisplatin-mediated ROS accumulation. (**E**–**H**) C6 cells were treated with cisplatin in the presence of small molecule inhibitors. Under all conditions, cisplatin induced a significant increase in ROS relative to vehicle-treated cells (p < 0.001). The protective effect of CYM-5478 is abolished by co-administration of either (**E**) S1P_2_ agonist, JTE-013, or (**F**) ROCK inhibitor, Y-27632. The CYM-5478-mediated attenuation of ROS accumulation can be phenocopied by treatment of C6 cells with either (**G**) Rac1 inhibitor, NSC23766, or (**H**) NADPH oxidase inhibitor, DPI. (N = 3) (**I**) Schematic representation of the likely signaling pathway by which S1P_2_ attenuates ROS accumulation. (*p < 0.05, **p < 0.01, ***p < 0.001).

To determine whether the cytoprotective effect of CYM-5478 was the result of decreased ROS production, we evaluated the sensitivity of C6 cells to cisplatin in the presence of a potent antioxidant, N-acetylcysteine^31^. When 1 mM N-acetylcysteine was added to culture media, there was no significant difference in the EC_50_ of cisplatin toward vehicle-treated or CYM-5478-treated cells (Fig. 4A), thus implicating ROS in the previously observed effect (Fig. 3C). To confirm that CYM-5478 is inhibiting the production of endogenous ROS, and is not directly acting as an antioxidant, we evaluated the ability of CYM-5478 to protect C6 cells from exogenously administered ROS. C6 cells were equally sensitive to hydrogen peroxide in the presence and absence of CYM-5478 (Fig. 4B), indicating that CYM-5478 does not have significant antioxidant activity, consistent with its actions via S1P_2_. Using CellROX® reagent, we confirm that there is an increase in ROS in C6 cells that are exposed to cisplatin (20 μM) for 24 hours (Fig. 4C), and that this increase can be significantly attenuated by co-administration of CYM-5478 (10 μM) (Fig. 4D). Treatment of C6 cells with either JTE-013 (S1P_2_ antagonist) (Fig. 4E) or Y-27632 (Rho-associated protein kinase (ROCK) inhibitor) (Fig. 4F) had no effect on cisplatin-induced ROS, but resulted in a complete attenuation of CYM-5478 activity. This demonstrates that the protective effect of CYM-5478 is mediated by S1P_2_ and activation of Rho signaling. Furthermore, we show that the effect of CYM-5478 can be phenocopied by treatment with either NSC23766 (Rac1 inhibitor) (Fig. 4G) or diphenyleneiodonium (DPI, NADPH oxidase inhibitor) (Fig. 4H). This implicates Rac1 and NOX as likely targets that are inhibited by CYM-5478.

## Discussion

The present study sought to identify cell-intrinsic phenomena that contribute to the cochlear degeneration that occurs in *S1pr2*^−/−^ mice. We found that loss of S1P_2_ results in the accumulation of ROS. The Nox family of NADPH oxidases are unusual in that they produce ROS as their primary function rather than as a byproduct^32^, and are thus a major source of signalling ROS. It is not surprising, then, that the activity of these enzymes requires strict control by a regulatory complex, with subunits that include known second-messengers such as Rac1. This provides a mechanism by which receptor-mediated signal transduction can limit ROS generation. Our current study demonstrates for the first time that S1P_2_ activity inhibits NOX3, resulting in the reduction of cytotoxic ROS accumulation. It is likely that this occurs via activation of Rho^26^, and subsequent inhibition of Rac^33^, which is an obligate member of the NOX complex^2^.

Interestingly, several previous studies have found that S1P signalling can regulate NOX activity, but possible mechanisms are poorly characterized and often paradoxical. For example, while S1P signalling has been shown to increase ROS in fibroblasts^9^, vascular endothelial cells^34^, and isolated arteries^8^, S1P signalling has been shown to decrease ROS accumulation in vascular smooth muscle cells^35^. This apparent inconsistency in the literature is likely due to the heterogeneity of S1P signaling, much of which stems from differential receptor expression coupled with the fact that different S1P receptor subtypes activate different downstream signalling pathways. It is particularly notable that S1P_1_ exclusively activates G_αi_^26^, which is a known activator of Rac1, whereas S1P_2_ is a strong inducer of G_α12/13_, which activates Rho^26^. Therefore, S1P_1_ would be expected to activate NOX, whereas S1P_2_ should inhibit it (Fig. 4I). Furthermore, it was recently demonstrated that S1P complexed with high-density lipoprotein (ApoM) acts as a biased agonist, inducing distinctly different S1P_1_-mediated effects compared to albumin-bound S1P^36^. This implies that ligand presentation can affect S1P signaling, and identifies an additional variable that may contribute to S1P otoprotective effects.

To underscore the translational relevance of this study, we have validated the activity of a recently identified S1P_2_ agonist, CYM-5478. This compound (PubChem SID #46371153, CID #7802604) was identified in a high throughput screen for S1P_2_ agonism and was used for SAR studies^27^, but was not systematically evaluated for receptor selectivity. In the current study, we provide the first demonstration that CYM-5478 is a potent and highly selective agonist for S1P_2_. In addition, we have used CYM-5478 to demonstrate that S1P_2_ mediates pro-migratory responses in oral squamous cell carcinoma (Patmanathan, manuscript under review), further demonstrating that CYM-5478 is a valuable tool for identifying biological functions of S1P_2_.

Interestingly, a recent study reports the identification of an autosomal-recessive nonsyndromic hearing impairment (ARNSHI) locus that contains the S1P_2_ gene (*S1PR2*)^37^. The authors further identify two mis-sense mutations that co-segregate with profound hearing loss in consanguineous families. This provides compelling evidence that the *S1pr2*^−/−^ mouse is a faithful model for the role of S1P signalling in hearing loss.

These combined results suggest that S1P_2_ represents a potential drug target for the prevention of the ototoxicity caused by cisplatin. Despite the 75–100% occurrence of hearing loss associated with cisplatin therapy^38^, platinum-based chemotherapeutics remain first-line treatments for lung cancer and other tumor types. It is well-established that toxic accumulation of ROS is at least partly responsible for the effect of cisplatin on hearing loss^39^. Interestingly, the use of antioxidant therapy has shown some otoprotective potential, but has had limited success in actual clinical studies^40^. There are a number of issues that may be complicating this approach. For example, antioxidant therapy is non-selective, and typically has a protective effect on tumor cells, thus interfering with the desired effect of cisplatin treatment. This can be partially ameliorated by careful adjustment of the dosing schedule^39^, or by transtympanic administration of the antioxidant^41^. As a targeted potential therapy, administration of an S1P_2_ agonist would be predicted to have increased selectivity for otoprotection, without known mechanisms for increasing tumor resistance to cisplatin. Furthermore, since S1P_2_ agonists would initially act extracellularly via cell-surface receptors to minimize endogenous ROS production, they should have higher potency and more favorable pharmacokinetics than antioxidants that must enter the cell and act on ROS that have already accumulated.

Further validation of the value of S1P_2_ as a therapeutic target was recently provided using an *ex vivo* culture model of gentamycin ototoxicity^42^. The authors demonstrated that administration of an antagonist for S1P_2_, but not S1P_1_ and S1P_3_, results in increased gentamycin ototoxicity^43^. They further show that inhibition of sphingosine kinase potentiates cisplatin ototoxicity and itself promotes apoptosis and hair cell loss^44^, which confirms the importance of endogenous S1P signalling in cochlear integrity. Additionally, S1P signalling has been implicated in other cellular and physiological functions in the inner ear^45^. Notably, S1P signalling has been shown to regulate cochlear blood flow and affect the integrity of the stria vascularis^20^,^46^. Future *in vivo* studies are required to further characterize S1P_2_ as a *bona fide* drug target for otoprotective therapy.

## Methods

### Materials

CYM-5478 (catalog #EN300-57094) was obtained from Enamine LLC. D-erythro-S1P (catalog #73914), N-acetylcysteine (catalog #A7250), hydrogen peroxide (catalog #216763), DPI (catalog #D2926), JTE-013 (catalog #J4080), and cisplatin (cis-Diammineplatinum(II) dichloride, catalog #P4394) were obtained from Sigma-Aldrich. Fluorescein phalloidin (catalog #F432), propidium iodide (catalog #P3566), and Hoechst 33342 (catalog #H3570) were obtained from Thermo Fisher Scientific. NSC23766 (item #13196) and Y-27632 (item #10005583) were obtained from Cayman Chemicals.

### Animal Welfare and Ethical statement

Mice were housed in ventilated cages in the vivarium at The Scripps Research Institute (TSRI) on a 12 hour light/12 hour dark cycle, with ad libitum access to water and standard chow. *S1pr2*^−/−^ mice were generated and maintained as described^47^ in a 129/SvJ, C57BL/6N mixed background. No procedures were performed on live animals for this study. All procedures were in compliance with state and federal regulations regarding animal welfare, followed the ARRIVE guidelines of the National Centre for the Replacement Refinement and Reduction of Animals in Research, and were performed as humanely as possible. All procedures were approved the Institutional Animal Care and Use Committee at TSRI and complied with the US National Research Council’s “Guide for the Care and Use of Laboratory Animals,” and the US Public Health Service’s “Policy on Humane Care and Use of Laboratory Animals” and “Guide for the Care and Use of Laboratory Animals”.

### Nitro blue tetrazolium (NBT) assay

Accumulation of ROS *ex vivo* was determined using a modification of a NBT assay previously described for visualization of ROS in cultured cells *in situ*^48^, in which the presence of ·O_2_^−^ is detected by the conversion of NBT to a blue, insoluble formazan precipitate^49^. Cochlea were rapidly isolated and exposed by perforating the boney wall, washed in HBSS, incubated with NBT (1.6 mg/ml) in HBSS at 37 °C for precisely 45 min, then fixed in 100% methanol and photographed with a stereo microscope equipped with a Nikon Coolpix 950 camera.

### Cell culture

C6 rat glioma cells (ATCC #CCL-107), MDA-MB-231 cells (ATCC #HTB-26), and HEK293 cells (ATCC #CRL-1573) were maintained as a monolayer culture on tissue culture dishes at 37 °C, 5% CO_2_, 100% humidity in Dulbecco’s modified Eagle’s medium supplemented with 10% heat-inactivated fetal bovine serum and antibiotics. S1P and CYM-5478 were solubilized with bovine serum albumin (0.1% final concentration) prior to treatment.

### NOX3 activity assay

NOX3 activity was determined as previously described^24^,^25^. Briefly, HEK293 cells were transfected with expression constructs for the NOX3 complex (*Nox3*, *NoxO1*, *NoxA1*), and co-transfected with the indicated S1P receptor, incubated for 16 hours, then replated in 96-well plates in Hank’s Balanced Saline Solution. Cells were treated with S1P (1 μM) or vehicle, and immediately evaluated for ROS by luminol assay. Indicated values were taken 15 minutes following stimulation.

### Receptor internalization assay

Cells were cultured on collagen-coated coverslips (cat #08-115 Millipore), transfected with S1P receptor-enhanced green fluorescence protein (EGFP) fusion constructs using Lipofectamine 3000 reagent (cat #L3000001, Thermo Fisher), and serum-starved for 4 hours prior to ligand stimulation essentially as described^50^.

### TGFα-shedding assay

The TGFα-shedding assay was performed essentially as described^28^. Briefly, HEK293 cells were co-transfected with the indicated receptor expression construct and TGFα-alkaline phosphatase using Lipofectamine 2000 reagent (cat #11668019, Thermo Fisher), collected by trypsinization, washed with phosphate-buffered saline, and seeded into 96-well plates in Hank’s Balanced Saline Solution (HBSS). To improve assay sensitivity, S1P_1_ and S1P_2_ cells were co-transfected with G_αq/i1_ chimeric protein, and S1P_4_ and S1P_5_ were co-transfected with G_αq/16_, as previously described^28^. Cells were stimulated with ligand for 1 hour, then alkaline phosphatase activity was detected in cells and in the supernatant. Receptor activity (% shedding) was defined as alkaline phosphatase activity of the supernatant/total alkaline phosphatase activity (cells + supernatant). Data processing and statistical analyses were performed with GraphPad Prism 6.

### Cell rounding assay

Cell rounding was performed essentially as described^51^. MDA-MB-231 breast cancer cells were seeded on collagen-coated coverslips at 30–50% confluence, incubated overnight, and serum-starved for 4 hours. They were then pretreated with JTE-013 or vehicle for 15 minutes, and treated with S1P, CYM-5478, or vehicle. After 15 min, the cells were fixed and stained with fluorescein phalloidin and DAPI for cell morphology. The number of cells with retracted neurites and the number of total cells were counted in three separate fields for each sample, and the percentage of cells with retracted neurites was calculated.

### Reverse transcriptase polymerase chain reaction (RT-PCR)

Total RNA was isolated from C6 glioma cells using Trizol reagent (Life Technologies) per manufacturer’s instructions. Approximately 2 μg of each sample was primed with oligo-dT and random hexamer primers prior using Thermo Scientific Maxima First Strand cDNA synthesis kit (Life Technologies). For quantitative real-time RT-PCR, targets were amplified with Maxima SYBR Green/ROX qPCR Master Mix (Life Technologies) on an Applied Biosystems ViiA 7 Real-Time PCR System (Life Technologies) using gene-specific primer pairs (Table 1). Quantitation was determined with a standard curve analysis as described^52^.

### Viability assay

Cell viability was determined by 3-(4,5-dimethylthiazol-2-yl)-2,5-diphenyltetrazolium bromide (MTT) assay as described^13^. Briefly, C6 cells were seeded into 96-well plates at 20,000 cells/well, incubated overnight, serum-starved for 4 h, and treated for 72 hours with cisplatin in the presence of CYM-5478 or vehicle.

### Cell death assay

Cell death was evaluated by propidium iodide exclusion assay. Cells were plated in 96-well plates at ~50% confluence, incubated overnight, then treated with cisplatin (67 μM) for 24 hours in the presence of vehicle or 10 μM CYM-5478. Cell were treated with Hoechst 33342 (1 mg/ml) and propidium iodide (0.3 mg/ml) for 20 minutes, washed with phosphate-buffered saline, then rapidly photographed with a Cytation 3 automated imager (Biotek Instruments, Inc.). % cell death reflects a ratio of propidium iodide-positive and Hoechst 33342-positive cells.

### Caspase assay

Cells were plated in clear-bottom, black-walled 96-well plates at ~50% confluence, incubated overnight, then treated overnight with cisplatin in the presence of vehicle or 10 μM CYM-5478. Caspase activation was evaluated by Caspase-Glo 3/7 Assay System (Promega Corporation) per manufacturer’s instructions.

### ROS assay

Cells were plated in clear-bottom, black-walled 96-well plates at ~50% confluence, incubated overnight, then treated overnight with cisplatin in the presence of the indicated compounds. All conditions were vehicle-controlled (0.1% DMSO). Cells were treated with CellROX® Orange reagent (ThermoFisher #C10443) per manufacturer’s protocol and counter stained with Hoechst 33342 (ThermoFisher #62249). Cells were photographed with a 20X objective lens, 3 fields per well, 3 wells per condition. Quantification was performed using ImageJ software, but dividing the total integrated density of CellROX® labelling by the number of cells per Hoechst staining.

## Additional Information

**How to cite this article**: Herr, D. R. *et al.* Sphingosine 1-phosphate receptor 2 (S1P_2_) attenuates reactive oxygen species formation and inhibits cell death: implications for otoprotective therapy. *Sci. Rep.* **6**, 24541; doi: 10.1038/srep24541 (2016).

## Figures and Tables

**Table 1:** Primers used for RT-PCR.

Gene	Forward primer	Reverse primer
*Gapdh*	AGTCTACTGGCGTCTTCACC	CCACGATGCCAAAGTTGTCA
*S1pr1*	GCATTGTCAAGCTCCCAGAG	GAAGAAATGGAGGGTGGGGA
*S1pr2*	CTGCTAGAGAGATGGACGGG	TCTTTAAGGCGGAGGTGTGT
*S1pr3*	GTCTCCACAGGTCAAGCTCT	CGGGCTGAAATGTATCGGTG
*S1pr4*	TGTGCTCTTTTGTGTGGTGG	CCAAGATCATCAGCACGGTG
*S1pr5*	CTCCAACAGTTTGCAGCGAT	TGGGAAGCGTCAGTCTGTAG
